# Antinociceptive compounds and LC-DAD-ESIMS^n^ profile from *Dictyoloma vandellianum* leaves

**DOI:** 10.1371/journal.pone.0224575

**Published:** 2019-10-29

**Authors:** Lucas Silva Abreu, Iura Muniz Alves, Renan Fernandes do Espírito Santo, Yuri Mangueira do Nascimento, César Augusto Gonçalves Dantas, Gisele Graça Leite dos Santos, Mireille Le Hyaric, Maria Lenise Silva Guedes, Marcelo Sobral da Silva, Cristiane Flora Villarreal, Eudes da Silva Velozo, Josean Fechine Tavares

**Affiliations:** 1 Programa de Pós-Graduação em Produtos Naturais e Sintéticos Bioativos, Universidade Federal da Paraíba, João Pessoa, Brazil; 2 Faculdade de Farmácia, Universidade Federal da Bahia, Salvador, Brazil; 3 Instituto de Química, Universidade Federal da Bahia, Salvador, Brazil; 4 Instituto Gonçalo Moniz, Fundação Oswaldo Cruz, Salvador, Brazil; 5 Departamento de Química, Universidade Federal de Juiz de Fora, Juiz de Fora, Brazil; 6 Instituto de Biologia, Universidade Federal da Bahia, Salvador, Brazil; Institute for Biological Research, SERBIA

## Abstract

Limonoids, quinolone alkaloids and chromones have been reported as constituents of *Dictyoloma vandellianum* Adr. Juss. (Rutaceae). Although those compounds are known for their biological activities, only the anti-inflammatory activity of chromones isolated from the underground parts has been evaluated. There are no studies of the pharmacological properties of the aerial parts of *D*. *vandellianum*. The present study was carried out to determine the phytochemical profile and antinociceptive activity of the methanol extract, fractions and isolated compounds of leaves of *D*. *vandellianum*. The phytochemical profile was performed by HLPC-DAD-ESIMS^n^ and pure substances obtained were characterized by MS and NMR spectroscopy. The antinociceptive activity was assessed using the formalin assay in mice, and the motor function in the rotarod test. ME and all the fractions obtained from ME produced antinociceptive effects. Among them, the ethyl ether fraction was the most active. Data from HPLC-DAD-ESIMS^n^ showed that the ethyl ether fraction presented 42 compounds. The major compounds isolated from this fraction—gallic acid, methyl gallate and 1,2,6-tri-O-galloyl-β-d-glucopyranose–were tested and produced antinociceptive effects. Gallic acid, methyl gallate and 1,2,6-tri-O-galloyl-β-d-glucopyranose at antinociceptive doses did not affect the motor performance in mice in the rotarod test. This work is the first report of the occurrence of gallotanins in *D*. *vandellianum*. In addition, the pharmacological study showed that *D*. *vandellianum* leaves present antinociceptive activity, probably induced by gallic acid, methyl gallate and 1,2,6-tri-O-galloyl-β-d-glucopyranose.

## Introduction

Pain is an unpleasant sensation that affects 20% of adults worldwide and it is associated with a wide range of diseases and tissue damage [1]. Currently, pharmacological management of pain is performed by drugs that are often not able to completely alleviate pain and induce frequent adverse effects [2]. In fact, all currently available drugs present poor analgesic efficacy in around half of treated chronic pain patients, stressing the importance of the development of new analgesics [3]. Historically, natural products have been a relevant source of chemical entities with analgesic properties, which can be clearly demonstrated by the key role of alkaloid morphine, and other opioids, in contemporary therapeutics [4]. Widespread use of anti-inflammatory analgesics, a class originating from the natural compound salicylic acid, also illustrates vast contribution of natural molecules to the pharmacotherapy of pain. Based on this, bioactive compounds of natural origin have been considered strategic options in the drug discovery process for analgesics [5].

Family Rutaceae includes about 160 genera and 1900 species, with a wide array of secondary chemical compounds presenting biological activities, such as coumarins [6–8], flavonoids [9–11] and alkaloids [12]. The genus *Dictyoloma* contains two species, *Dictyoloma peruvianum* Planch., which occurs in Peru and Bolivia, and *Dictyoloma vandellianum* Adr. Juss., popularly known as “tingui-preto” in Brazil [13]. Previous phytochemical studies of the fruits, stem, leaves and roots of *D*. *vandellianum* have led to the isolation of several limonoids, quinolone alkaloids and chromones [14–17]. These classes of compounds display a wide range of biological effects, such as anti-tumor, anti-malarial, anti-leishmania, anti-microbial, anti-inflammatory and anti-viral [17–22]. Recently, relevant anti-inflammatory properties of chromones isolated from *D*. *vandellianum* were demonstrated. Chromones exhibit in vitro and in vivo anti-inflammatory effects, probably due to glucocorticoid receptor activation and inhibition of the transcriptional activity of NF-κB [23]. Considering that anti-inflammatory compounds frequently also exhibit analgesic properties, the present work was designed to investigate the hypothesis that *D*. *vandellianum* has constituents with antinociceptive action. Therefore, the phytochemical profile of *D*. *vandellianum* was obtained by HPLC-DAD-ESIMS^n^ and extract, fractions and isolated compounds were tested to antinociceptive effects in the formalin assay. This test, described by Dubuisson and Denis in 1977 [24], is one of the most valuable and widely used nociception assays in the preclinical identification of compounds with analgesic potential [25]. The formalin test is a model of pain with two distinctive phases that may indicate different types of pain. The early phase is a result of direct stimulation of nociceptors, while the late phase is caused by local inflammation with a release of inflammatory and hyperalgesic mediators [26,27]. These two phases of formalin test have obvious differential mechanisms, and therefore this test is useful not only for assessing analgesic effect of substances, but also for elucidating the mechanism of analgesia [27]. On the other hand, although the behavioral response in the formalin test reflects changes in nociception, other factors that influence behavior, such as changes in motor function, may affect this response [25]. Therefore, the motor performance of mice was also evaluated by using the rotarod test, in order to corroborate the antinociceptive properties demonstrated in the formalin assay.

## Materials and methods

### General

Nuclear magnetic resonance ^1^H and ^13^C (single and two-dimensional) spectra were obtained on Varian spectrometer, model Gemini-500 (Varian, Inc., Palo Alto, CA, USA) (^1^H: 500 MHz and ^13^C: 125 MHz); using CD_3_OH solvent and TMS as internal standard.

All chromatographic separations were performed on octadecyl-functionalized 200–400 mesh (Sigma-Aldrich), using opened columns (40 × 4.5 cm, 45 × 5 cm, 15 × 8 cm). Thin-layer chromatography (TLC) was performed on glass plates covered with silica gel PF_254_ Merck 60 and revealed with iodine vapor and/or UV light (254 and 365 nm).

All solvents (analytical/HPLC grade) were purchased from Quimex (São Paulo, Brazil) and Tedia (Rio de Janeiro, Brazil) and used without further purification. Indomethacin and dimethyl sulfoxide were obtained from Sigma Chemical Company (St. Louis, MO, USA). Indomethacin was dissolved in Tris HCl 0.1 M pH 8.0 plus saline. The chloroform fractions were dissolved in 5% DMSO plus saline, and the remaining fractions and compounds were dissolved directly in saline.

### Plant material

The specimens were collected in March 2005 in Piatã (Inubia district, Bahia, Brazil), at an altitude of about 1304 m above mean sea level. Access registration in the National Management System of Genetic Patrimony and Associated Traditional Knowledge (SISGEN) was obtained under number A737BB9. The plant species were identified by Prof. Maria Lenise Silva Guedes, and deposited at the Herbarium Alexandre Leal Costa (ALCB), Institute of Biology, Federal University of Bahia with the registrations 69,163 (13°14′43″S, 41°45′28″W) and 88,951 (13°04′25″S, 41°04′51″W). The dried leaves and extracts were stored in a freezer at −8 °C until used.

### Preparation of extracts, fractions and isolation of compounds

The leaves (1.2 kg) from *D*. *vandellianum* were macerated three times in methanol (6 L) for 7 days. The combined extracts were concentrated under reduced pressure, suspended in water (500 mL) and extracted successively with chloroform (5 × 150 mL), ethyl ether (5 × 150 mL) and ethyl acetate (5 × 150 mL). An aliquot (4 g) of the ether fraction (22 g) was subjected to a C_18_ reversed-phase open-column chromatography using a gradient of water and methanol as eluent. Fractions of 100 mL were collected. Fractions 14, 23 and 28 resulted in the gallic acid (350 mg), methyl gallate (130 mg) and 1,2,6-tri-*O*-galloyl-β-d-glucopyranose (230 mg), respectively.

### Liquid chromatography-mass spectrometry instrumentation and conditions

A Shimadzu^®^ (Kyoto, Japan) High Performance Liquid Chromatography System, coupled with an Amazon X or micrOTOF II (Bruker Daltonics, Billerica, MA, USA) with an electrospray ion (ESI) source, was used to perform the ESI-MS^n^ and HRESIMS analysis, respectively. The LC System consisted of a LC-20AD solvent pump unit (flow rate of 600 μL.min^−1^); a DGU-20A_5_ online degasser; a CBM-20A system controller and a SPD-M20A (190–800 nm) diode array detector. The LC separation was performed on a Kromasil C-18 5 μm 100Å, 250 × 4.6 mm (Kromasil, Bohus, Sweden) analytical column. Injections (20 μL) were performed using an autosampler (SIL-10AF). The mobile phase consisted of 0.1% formic acid in water (solvent A) and methanol (solvent B). Exploratory linear gradient (5 × 100% B) was performed to elution in 90 min. The analysis parameters are as follows: capillary 4.5 kV, ESI in negative mode, final plate offset 500 V, 40 psi nebulizer, dry gas (N_2_) with flow rate of 8 mL/min and a temperature of 300 °C. CID fragmentation, in Amazon X, was achieved in auto MS/MS mode using enhanced resolution mode for MS and MS/MS mode. The spectra (*m*/*z* 50–1000) were recorded every 2 s.

### Animals

Experiments were performed on male Swiss Webster mice (20–25 g) obtained from the Animal Facilities of the Gonçalo Moniz Institute, FIOCRUZ. Mice were housed in temperature-controlled rooms (22–23 °C), under a 12:12 h light-dark cycle, with access to water and food ad libitum. Environmental enrichment was obtained with mouse igloos. Animal care and handling procedures were in strict accordance with the recommendations in the Guide for the Care and Use of Laboratory Animals of the National Institutes of Health. The present protocol was approved by the Institutional Animal Care and Use Committee, Ethics Committee for Animal Experimentation of FIOCRUZ (CEUA/FIOCRUZ. Permit Number: L-IGM-015/2013). Every effort was made to minimize the number of animals used and any discomfort. Accordingly, the animals were only used once and were sacrificed immediately after experimentation with isoflurane overdose. Behavioral tests were performed without knowing to which experimental group each mouse belonged.

### Antinociceptive activity—Formalin test

The mice were placed in an open Plexiglas observation chamber for 30 min to acclimate to their surroundings. They were then removed and gently restrained while 20 μL of 2.5% formalin (1:100 dilution of stock formalin solution, 37% formaldehyde in 0.9% saline) was injected subcutaneously into the dorsal surface of the hind paw using a 30 gauge needle. Following injection, the mice were returned to the observation chamber for a 30 min observation period. The nociceptive score was determined by counting the time the animal spent licking the injected paw during the early phase (0–10 min) and the late phase (10–30 min) [28]. The effects of ME (1.95–125 mg/kg) and its ether, ethyl acetate and chloroform fractions (100 mg/kg) were evaluated on the formalin test. Next, gallic acid, methyl gallate and 1,2,6-tri-*O*-galloyl-β-d-glucopyranose (0.19–200 mg/kg) obtained from the ethyl ether fraction were also evaluated. All treatments were administered once, by intraperitoneal route, 40 min before the injection of formalin. Indomethacin (10 mg/kg) and morphine (5 mg/kg) were used as the reference drugs. Vehicle group was treated with saline or 5% DMSO plus saline, as appropriate.

### Motor function assay—Rotarod test

To evaluate possible non-specific muscle-relaxant or sedative effects of the treatments, immediately before the formalin assay, the mice were submitted to the rotarod test, in a modified form as previously described [29]. The rotarod apparatus (Insight, Ribeirão Preto, SP, Brazil) consisted of a bar with a diameter of 3 cm, subdivided into five compartments. The bar rotated at a constant speed of eight revolutions per min. Mice were trained 24 h before the experiment to remain on the bar for 120 s. Those not remaining on the bar for two consecutive periods of 120 s were not included in the study. Forty minutes after the intraperitoneal injection of diazepam (10 mg/kg, reference drug), ME (200 mg/kg), gallic acid (200 mg/kg), methyl gallate (200 mg/kg), 1,2,6-tri-*O*-galloyl-β-d-glucopyranose (200 mg/kg) or vehicle, the animals were placed on the rotating rod and the latency to fall was measured for up to 120 s. The results are expressed as the average time(s) the animals remained on the rotarod in each group.

### Statistical analysis

Data are presented as means ± standard error of the means (SEM) of measurements made on six animals in each group. Comparisons between three or more treatments were made using one-way ANOVA with Tukey’s post hoc test. All data were analyzed using Prism 5 Computer Software (GraphPad, San Diego, CA, USA). Statistical differences were considered to be significant at *p* < 0.05. The ED_50_ (dose of an agonist that produces 50% of the maximal possible effect of that agonist) values were expressed with a confidence interval (CI). Individual dose–response curves were fitted with the Hill logistic equation. ED_50_ values were obtained as the dose at which a half maximal reduction in nociceptive score occurred, calculated with 95% confidence limits.

## Results

### Phytochemical study

**Fig 1:**
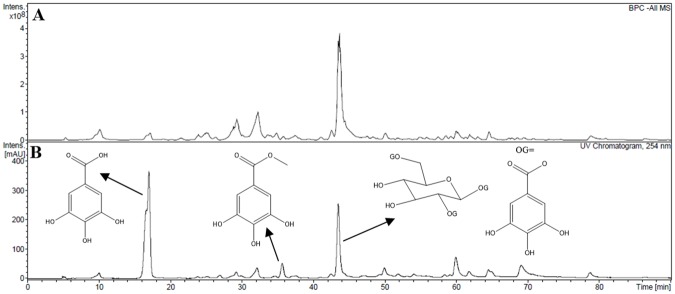
Base peak chromatograms (BPC) in negative ion mode (A) and DAD chromatogram at 254 nm (B) of ethyl ether fraction from leaves of *D*. *vandellianum* by HPLC-DAD-ESIMS^n^. In (B), the peaks corresponding to gallic acid, methyl gallate and 1,2,6-tri-O-galloyl-β-d-glucopyranose was demonstrated.

The HPLC-DAD-ESIMS^n^ analysis detected the presence of 42 compounds in the ether ethyl fraction (see Fig 1 and Table 1). The three known compounds that were isolated from this fraction had their structures confirmed by ^1^H and ^13^C NMR and mass spectrometry. The isolated compounds were identified as gallic acid, methyl gallate and 1,2,6-tri-O-galloyl-β-d-glucopyranose.

**Table 1:** Characterization of the compounds tentatively identified by HPLC-ESI-MS^n^ in *Dictyoloma vandelliaum*.

Peak No.	*t*_R_ (min.)	*m*/*z* [M − H]^−^	Molecular formula	Calcd.	eror (ppm)	MS^2^/MS^3^	Tentative assignment	Reference
1	5.3	331.0671	C_13_H_16_O_10_	331.0659	-3.4	MS^2^ [331]: 313 (7.63); 271 (51.21); 253 (3.47); 241 (3.82); 211 (28.15); 193 (38.24); 169 (100); 125 (13.75)	Galloylhexose I	[30,31]
2	10.1	355.0284	C_14_H_12_O_11_	355.0295	3.3	MS^2^ [355]: 337 (100); 293 (1.32); 249 (1.43)	Chebulic acid	[31]
3	16.7	169.0125	C_7_H_6_O_5_	169.0131	3.8	MS^2^ [169]: 125 (100); 81 (0.25)/MS^3^ [169 → 125]: 97 (73.07); 81 (100); 69 (17.81)	Galic acid^a^	[31]
4	17.9	343.0658	C_14_H_16_O_10_	343.0659	0.5	MS^2^ [343]: 191 (100); 169 (7.01)/MS^3^ [343 → 191]: 173 (84); 127 (100); 93 (62.43); 85 (78)	5-O-galloylquinic acid	[32]
5	18.8	331.0653	C_13_H_16_O_10_	331.0659	2.0	MS^2^ [331]: 271 (100); 211 (1.35); 169 (0.70)/MS^3^ [331 → 271]: 211 (100); 169 (8.45); 125 (2.08)	Galloylhexose II	[30,31]
6	20.1	331.0671	C_13_H_16_O_10_	331.0671	-3.4	MS^2^ [331]: 313 (1.90); 271 (100); 241 (1.53); 211 (1.79); 169 (1.4)/MS^3^ [331 → 271]: 211 (100); 169 (9.14); 125 (1.72)	Galloylhexose III	[30,31]
7	20.6	343.0671	C_14_H_16_O_10_	343.0659	-3.4	MS^2^ [343]: 191 (20.96); 173 (18.96); 169 (100); 125 (6.67)/MS^3^ [343 → 169]: 125 (100)	4-O-galloylquinic acid	[32]
8	22.7	483.0753	C_20_H_20_O_14_	483.0769	3.4	MS^2^ [483]: 331 (45.67); 313 (100); 271 (17.19); 241 (6.17); 211 (7.33); 193 (14.17)/MS^3^ [483 → 313]: 253 (8.22); 169 (100); 151 (8.44); 125 (7.21)	Digalloyl-hexoside I	[30,31]
10	25.5	483.0751	C_20_H_20_O_14_	483.0769	3.8	MS^2^ [483]: 331 (33.47); 313 (100); 271 (13.45); 211 (7.48); 193 (10.11)/MS^3^ [483 → 313]: 295 (4.46); 169 (100); 151 (7.10); 125 (16.19)	Digalloyl-hexoside II	[30,31]
11	26.3	495.0760	C_21_H_20_O_14_	495.0769	1.9	MS^2^ [495]: 477 (3.88); 343 (100); 325 (47.37); 245 (3.84); 193 (7.70)/MS^3^ [495 → 343]: 191 (27.41); 169 (100); 125 (8.13)	Digalloylquinic acid I	[32]
12	29.3	483.0749	C_20_H_20_O_14_	483.0769	4.2	MS^2^ [483]: 423 (92.69); 405 (16.88); 331 (17.16); 313 (69.26); 295 (18.25); 271 (100); 241 (24.9); 211 (63.65); 193 (50.25)/MS^3^ [483 → 271]: 253 (3.38); 211 (100); 193 (12.41); 169 (9.2); 125 (2.80)	Digalloyl-hexoside III	[30,31]
13	32.1	483.0745	C_20_H_20_O_14_	483.0769	5.0	MS^2^ [483]: 423 (92.28); 405 (22.22); 331 (14); 313 (68); 295 (24.61); 271 (100); 241 (23.71); 211 (79.58); 193 (61.39)/MS^3^ [483 → 271]: 253 (4.47); 211 (100); 193 (15.81); 169 (13.34); 125 (2.11)	Digalloyl-hexoside IV	[30,31]
14	34.8	495.0737	C_21_H_20_O_14_	495.0769	6.5	MS^2^ [495]: 343 (100); 325 (5.52); 191 (5.57)/MS^3^ [495 → 343]: 191 (100); 167 (90.45); 125 (5.55)	Digalloylquinic acid II	[32]
15	35.1	635.0880	C_27_H_24_O_18_	635.0878	-0.2	MS^2^ [635]: 483 (53.16); 465 (100); 313 (19.11); 271 (3.83)/MS^3^ [635 → 465]: 313 (100); 295 (11.42); 235 (8.74); 169 (9.84)	Tri-galloyl-hexoside I	[30]
16	35.8	183.0308	C_8_H_8_O_5_	183.0297	-4,9	MS^2^ [183]: 168 (100); 124 (68)	Methylgallate^a^	[30]
17	36.1	483.0760	C_20_H_20_O_14_	483.0769	1.9	MS^2^ [483]: 465 (18.8); 439 (14.74); 423 (43.94); 331 (12.62); 313 (28.82); 271 (100); 241 (4.5); 211 (51.02); 193 (53.30)/MS^3^ [483 → 271]: 253 (1.89); 211 (100); 169 (11.26); 125 (2.56)	Digalloyl-hexoside V	[30,31]
18	37.3	495.0746	C_21_H_20_O_14_	495.0769	4.7	MS^2^ [495]: 343 (100); 325 (23.17); 289 (3.73); 245 (2.03); 193 (13.47)/MS^3^ [495 → 343]: 191 (13.22); 169 (100); 125 (7.58)	Digalloylquinic acid III	[32]
19	37.3	635.0848	C_27_H_24_O_18_	635.0878	4.9	MS^2^ [635]: 483 (50.15); 465 (100); 423 (2.73); 313 (20.03); 297 (2.41); 271 (3.71); 251 (1.24)/MS^3^ [635 → 465]: 313 (100); 295 (16.37); 169 (29.37)	Tri-galloyl-hexoside II	[30]
20	38.6	321.0235	C_14_H_10_O_9_	321.0241	1.9	MS^2^ [321]: 169 (100); 125 (9.36)/MS^3^ [321 → 169]: 125 (100)	Digallic acid	[30]
21	39.9	321.0235	C_14_H_10_O_9_	321.0241	1.9	MS^2^ [321]: 169 (100); 125 (9.36)/MS^3^ [321 → 169]: 125 (100)	Digallic acid II	[30]
22	42.5	647.0855	C_28_H_24_O_18_	647.0878	3.7	MS^2^ [647]: 495 (100); 477 (10.15); 343 (16.29); 325 (8.14); 307 (1.06)/MS^3^ [647 → 495]: 477 (3.11); 343 (100); 325 (43.34); 307 (3.08); 289 (3.19); 245 (2.83); 193 (5.84)	3.4.5-tri-O-galloylquinic acid	[32]
23	43.6	635.0890	C_27_H_24_O_18_	635.0878	-1.7	MS^2^ [635]: 483 (82.99); 465 (100); 423 (8.61); 313 (41.91); 295 (9.50); 271 (11.02)/MS^3^ [635 → 465]: 313 (100); 295 (12.96); 169 (11.64)	1,2,6-tri-O-galloyl-β-d-glucopyranose ^a^	[30]
24	49.2	197.0451	C_9_H_10_O_5_	197.0444	-3.3	MS^2^ [197]: 169 (100); 125 (9.88)/MS^3^ [197 → 169]: 125 (100)	Ethyl gallate	[33]
25	50.0	953.0927	C_41_H_30_O_27_	953.0890	-3.8	MS^2^ [953]: 935 (21); 801 (7.45); 633 (17.85); 615 (10.65); 589 (7.38); 481 (10.9); 463 (100)	Chebulagic acid	[31]
26	51.2	787.1050	C_34_H_28_O_22_	787.0988	-7.8	MS^2^ [787]: 635 (100); 617 (62.74); 483 (9.37); 465 (26.41); 313 (4.3)/MS^3^ [787 → 635]: 483 (41.72); 465 (100); 313 (57.95)	Tetra-*O*-galloylhexoside	[30]
27	52.8	635.0906	C_27_H_24_O_18_	635.0878	-4.3	MS^2^ [635]: 577 (9.3); 483 (66.3); 465 (100); 423 (10.36); 313 (41.42); 271 (6.98)/MS^3^ [635 → 465]: 447 (4.15); 313 (100); 295 (8.7); 169 (7.23)	Tri-galloyl-hexoside IV	[30]
28	56.7	447.0931	C_21_H_20_O_11_	447.0921	-2.0	MS^2^ [447]: 429 (18.9); 357 (72.2); 327 (100)/MS^3^ [447 → 327]: 299 (100); 284 (14.53); 191 (2.23)	Isoorientin	[34]
29	57.4	635.0883	C_27_H_24_O_18_	635.0878	-0.6	MS^2^ [635]: 599 (33); 483 (74.18); 465 (100); 313 (35.37); 301 (5.28); 271 (10)/MS^3^ [635 → 465]: 447 (5.74); 313 (100); 295 (12.61); 169 (3.68)	Tri-galloyl-hexoside V	[30]
30	58.5	431.1002	C_21_H_20_O_10_	431.0972	-6.8	MS^2^ [431]: 341 (7.93); 311 (100); 283 (4.91)/MS^3^ [431 → 311]: 283 (100); 191 (1.52)	Vitexin	[34]
31	60.8	615.1010	C_28_H_24_O_16_	615.0980	-4.8	MS^2^ [615]: 463 (100); 301 (43.71); 271 (2.5)/MS^3^ [615 → 463]: 301 (100); 271 (1.9)	Galloylquercetin hexoside	[34]
32	61.9	583.1111	C_28_H_24_O_14_	583.1082	-4.9	MS^2^ [583]: 431 (100); 413 (23.24); 311 (10.88); 293 (5.64)/MS^3^ [583 → 431]: 341 (8.91); 311 (100); 283 (7.04)	(Iso)vitexin galloyl	[34]
33	61.9	431.0984	C_21_H_20_O_10_	431.0972	-2.6	MS^2^ [431]: 413 (7.23); 341 (37.47); 311 (100); 283 (2.25)/MS^3^ [431 → 311]: 283 (100)	Isovitexin	[34]
34	64.6	583.1113	C_28_H_24_O_14_	583.1082	-5.3	MS^2^ [583]: 431 (100); 413 (11.98); 311 (4.73)/MS^3^ [583 → 431]: 341 (27.95); 311 (100); 283 (3.4)	(Iso)vitexin galloyl	[34]
35	65.1	463.0893	C_21_H_20_O_12_	463.0871	-4.7	MS^2^ [463]: 301 (100); 271 (2.53); 179 (3.27)/MS^3^ [463 → 301]: 271 (81.54); 255 (54.71); 179 (100); 151 (83.54)	Quercetin-*O*-hexoside	[35]
36	66.0	599.1075	C_28_H_24_O_15_	599.1031	-7.3	MS^2^ [599]: 447 (17.67); 357 (9.74); 327 (14.52); 313 (27.64); 285 (100); 271 (2.19)/MS^3^ [599 → 285]: 217 (86.4); 199 (64.36); 175 (100); 151 (41.27)	Astragalin-O-gallate I	[33]
37	67.3	599.1040	C_28_H_24_O_15_	599.1031	-1.4	MS^2^ [599]: 447 (79.39); 357 (4.12); 327 (6.12); 313 (100); 285 (78.65); 271 (3.04)/MS^3^ [599 → 313]: 241 (16.56); 169 (100); 125 (21.48)	Astragalin-O-gallate II	[33]
38	69.2	301.0026	C_14_H_6_O_8_	301.0037	3.9	MS^2^ [301]: 257 (100); 229 (88.42); 185 (63.06); 157 (5.74)/MS^3^ [301 → 257]: 229 (83.14); 213 (13.31); 201 (12.95); 185 (100); 157 (3.98)	Ellagic acid	[36]
39	70.8	447.0931	C_21_H_20_O_11_	447.0921	-2.0	MS^2^ [447]: 285 (100); 255 (46); 227 (8.7); 179 (1.43)/MS^3^ [477 → 285]: 267 (10.26); 255 (100); 227 (15.75)	Kaempferol-*O*-hexoside	[35]
40	78.8	301.0351	C_15_H_10_O_7_	301.0342	-2.7	MS^2^ [301]: 273 (18.47); 179 (100); 151 (84.4)/MS^3^ [301 → 179]: 169 (20.13); 151 (100)	Quercetin	[37]
41	80.9	593.1295	C_30_H_26_O_13_	593.1289	-0.9	MS^2^ [593]: 447 (11.79); 285 (100)/MS^3^ [593 → 285]: 257 (100); 185 (31.12); 151 (73.39)	Tribuloside	[33]
42	86.4	285.0404	C_15_H_10_O_6_	285.0393	-3.6	MS^2^ [285]: 257 (67.73); 243 (77.95); 229 (100); 185 (78.03); 169 (72.37); 151 (52.27)	Kaempferol	[30]

^I, II, III, IV and V^ Numbers used to discriminate putative individual isomers.

^a^ Identified by ^1^H and ^13^C NMR

### Antinociceptive activity

**Fig 2:**
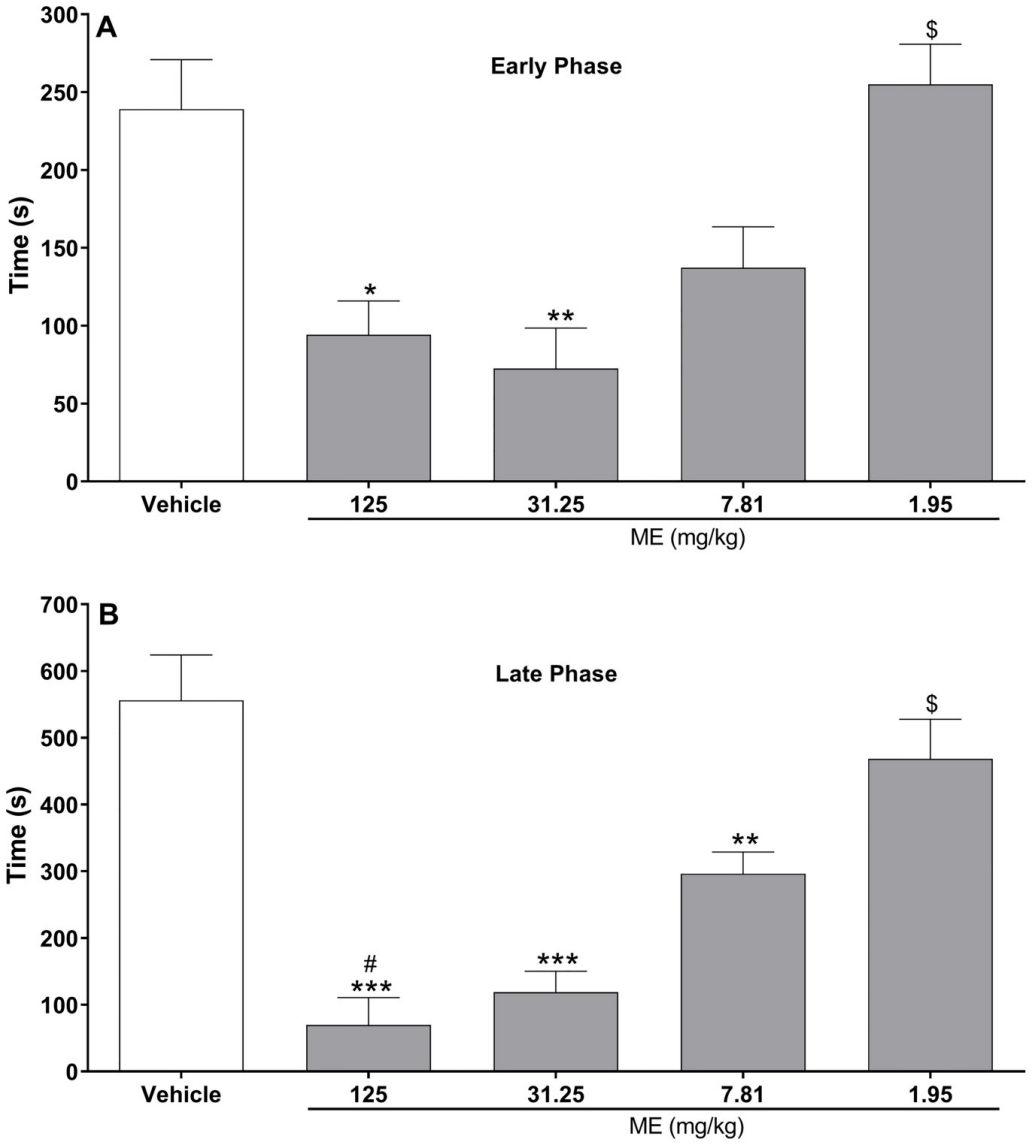
Effects of the methanol extract of *D*. *vandellianum* leaves (ME) on formalin test in mice. Mice were treated with vehicle (saline, control group) or ME (125–1.95 mg/kg) by intraperitoneal route 40 min before the intraplantar injection of formalin (injected at time zero). Mice were observed from 0 to 10 min (early phase; **A**) and from 10 to 30 min (late phase; **B**), and a nociceptive score was determined for each period by counting the time in seconds that the animal spent licking the injected limb during the observation time. Data are expressed as mean times ± S.E.M.; *n* = 6 mice per group. Statistical significance relative to the control group: *(*p* < 0.05); **(*p* < 0.01); ***(*p* < 0.001). Statistical significance relative to the 7.81 mg/kg group: ^#^ (*p* < 0.05). Statistical significance relative to the 125 and 31.25 mg/kg groups: ^$^ (*p* < 0.05). ANOVA followed by Tukey’s test.

The antinociceptive properties of test compounds were investigated in the formalin test in mice. Initially, the effect of systemic injection of methanol extract of *D*. *vandellianum* leaves (ME) on the formalin-induced nociception was evaluated (Fig2). Administration of formalin in control mice induced a biphasic flinching response, with early phase ranging from 0 to 10 min and late phase from 10 to 30 min after the injection. Intraperitoneal injection of ME (7.8–125 mg/kg), 40 min prior to the formalin injection, produced a significant antinociceptive effect in both early and late phases of the test (Fig 2A and 2B, respectively). ME at 1.95 mg/kg did not induce antinociception in the formalin test. In the late phase of the test, the ME-induced antinociceptive effect showed a dose-dependent profile, with ED_50_ value of 8.03 mg/kg (CI 4.62 to 13.94).

**Fig 3:**
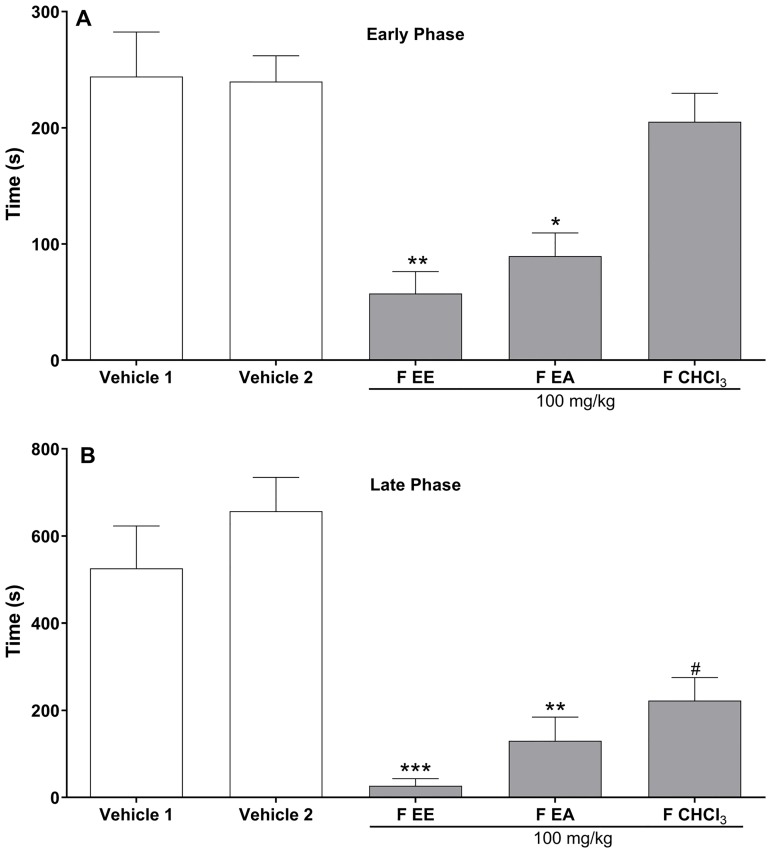
Effects of different fractions from methanol extract of *D*. *vandellianum* leaves (ME) on formalin test in mice. Mice were treated with vehicle (control groups) or fractions (100 mg/kg) by intraperitoneal route 40 min before the intraplantar injection of formalin (injected at time zero). FEE: ether fraction from ME (solubilized in saline); FEA: ethyl acetate fraction from ME (solubilized in 5% DMSO); FCHCl_3_: chloroform fraction from ME (solubilized in 5% DMSO). Vehicle 1: saline (control group of FEE). Vehicle 2: 5% DMSO plus saline (control group of FEA and FCHCl_3_). Mice were observed from 0 to 10 min (early phase; **A**) and from 10 to 30 min (late phase; **B**), and a nociceptive score was determined for each period by counting the time in seconds that the animal spent licking the injected limb during the observation time. Data are expressed as mean times ± S.E.M.; *n* = 6 mice per group. * Significantly different from vehicle 1 group (*p* < 0.05); **significantly different from vehicle 1 group (*p* < 0.01); *** significantly different from vehicle 1 group (*p* < 0.001); # significantly different from vehicle 2 group (*p* < 0.01). ANOVA followed by Tukey’s test.

Antinociceptive effects of the three fractions (chloroform, ethyl ether and ethyl acetate) obtained from ME were evaluated next (Fig 3). A decrease in nociceptive score was induced by the systemic injection of ethyl ether (FEE; 100 mg/kg) and ethyl acetate (FEA; 100 mg/kg) fractions in both early and late phases of formalin test. Inhibitory effect of the ethyl ether fraction was of 76% and 95% on early and late phases of formalin test, respectively. Ethyl acetate fraction induced an inhibitory effect of 63% and 75% on the early and late phases, respectively. On the other hand, the pretreatment with chloroform fraction (FCHCl_3_, 100 mg/kg) produced antinociceptive effect only in late phase of the test, displaying 66% of nociception inhibition.

**Fig 4:**
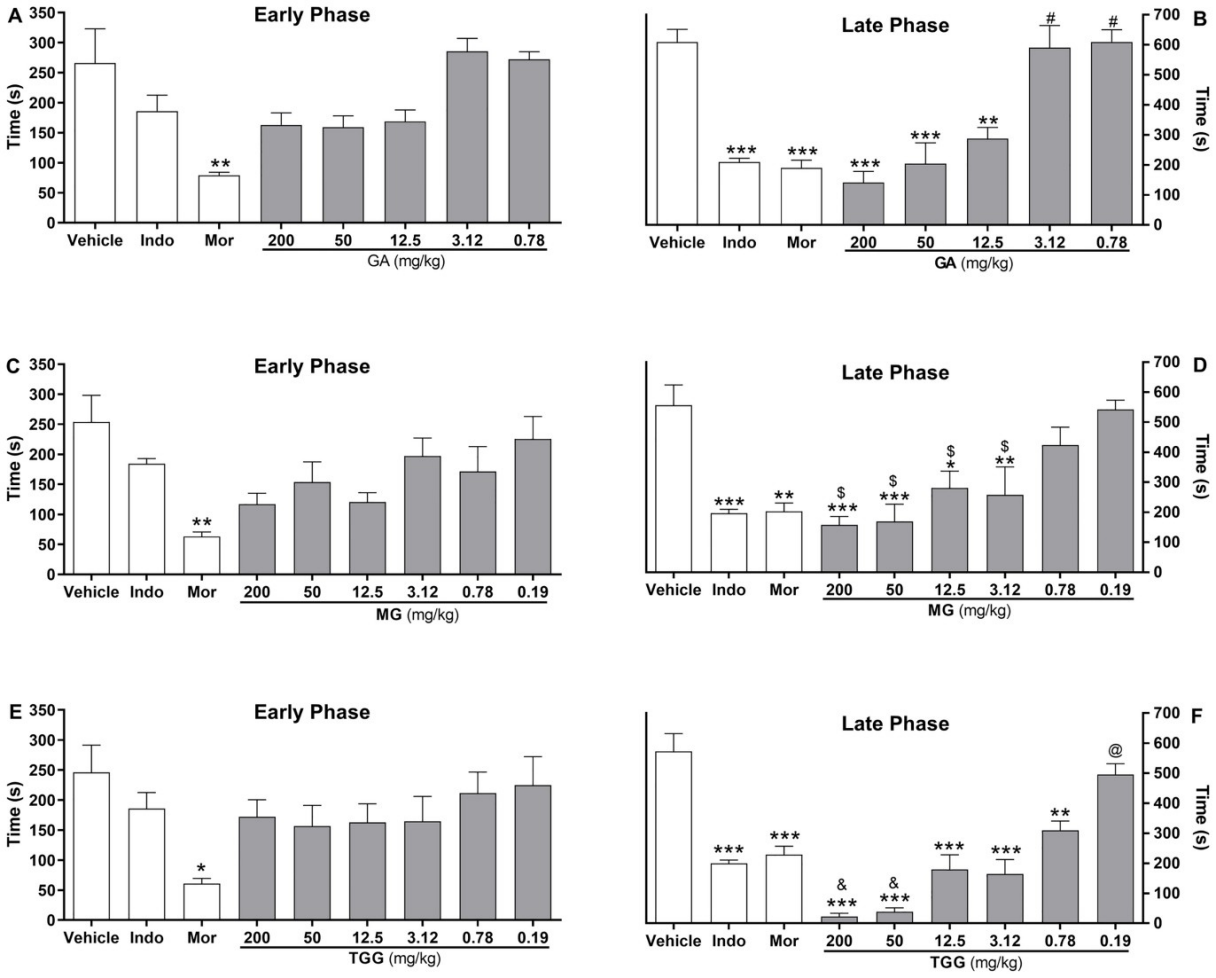
Effects of gallic acid, methyl gallate and 1,2,6-tri-O-galloyl-β-d-glucopyranose on formalin test in mice. Mice were treated with vehicle (saline, control group), indomethacin (Indo; 10 mg/kg, reference drug), morphine (Mor; 5 mg/kg, reference drug), gallic acid (GA; 0.78–200 mg/kg, panels A and B), methyl gallate (MG; 0.19–200 mg/kg, panels C and D) and 1,2,6-tri-O-galloyl-β-d-glucopyranose (TGG; 0.19–200 mg/kg, panels E and F) by intraperitoneal route 40 min before the intraplantar injection of formalin (injected at time zero). Mice were observed from 0 to 10 min (early phase, panels **A**, **C and E**) and from 10 to 30 min (late phase, panels **B**, **D and F**), and a nociception score was determined for each period by counting the time in seconds that the animal spent licking the injected limb during the observation time. Data are expressed as mean times ± S.E.M.; *n* = 6 mice per group. Statistical significance relative to the control group: *(*p* < 0.05); **(*p* < 0.01); ***(*p* < 0.001). Statistical significance relative to the 200, 50 and 12.5 mg/kg groups: ^#^ (*p* < 0.01). Statistical significance relative to the 0.78 mg/kg group: ^&^ (*p* < 0.001). Statistical significance relative to the 0.19 mg/kg groups: ^$^ (*p* < 0.05). Statistical significance relative to the lower doses: ^@^ (*p* < 0.05). ANOVA followed by Tukey’s test.

Considering that FEE induced a relevant antinociceptive effect in the formalin assay, its phytochemical profile was next evaluated to identify constituents within this fraction with antinociceptive properties. A phase-reverse chromatography of the fraction was performed, allowing isolation of gallic acid (GA), methyl gallate (MG) and 1,2,6-tri-O-galloyl-β-d-glucopyranose (TGG). GA at 12.5 (*p* < 0.01), 50 (*p* < 0.001) and 200 (*p* < 0.001) mg/kg, administered by intraperitoneal route 40 min before formalin injection, reduced the nociceptive behavior of mice in late, but not early phase of the test (Fig 4). GA at 3.12 and 0.78 mg/kg did not induce effect on formalin test. Pretreatment with indomethacin (10 mg/kg, ip), a standard nonsteroidal anti-inflammatory drug, produced a similar inhibition profile of late phase (*p* < 0.001). As expected, the pretreatment with morphine (5 mg/kg, ip), a gold standard opioid, inhibited both the early (*p* < 0.01) and late (*p* < 0.001) phase of the formalin test.

Intraperitoneal administration of MG (3.1–200 mg/kg), 40 min before formalin injection, inhibited the late phase of formalin test (*p* < 0.001; *p* < 0.01; *p* < 0.05). MG at 0.78 and 0.19 mg/kg showed no antinociceptive effect. MG, at all doses tested, did not induce a statistically significant reduction in the early phase of the formalin test. Systemic pretreatment with TGG at doses between 0.78 and 200 mg/kg inhibited the late, but not the early phase of the formalin test (*p* < 0.001; *p* < 0.01). TGG at 0.19 mg/kg had no effect in this assay. In the late phase of the formalin test, the TGG-induced antinociception was a dose-dependent effect, as indicated by the statistically significant difference between active doses (*p* < 0.01), displaying an ED_50_ value of 0.80 mg/kg (CI 0.22 to 2.57).

**Fig 5:**
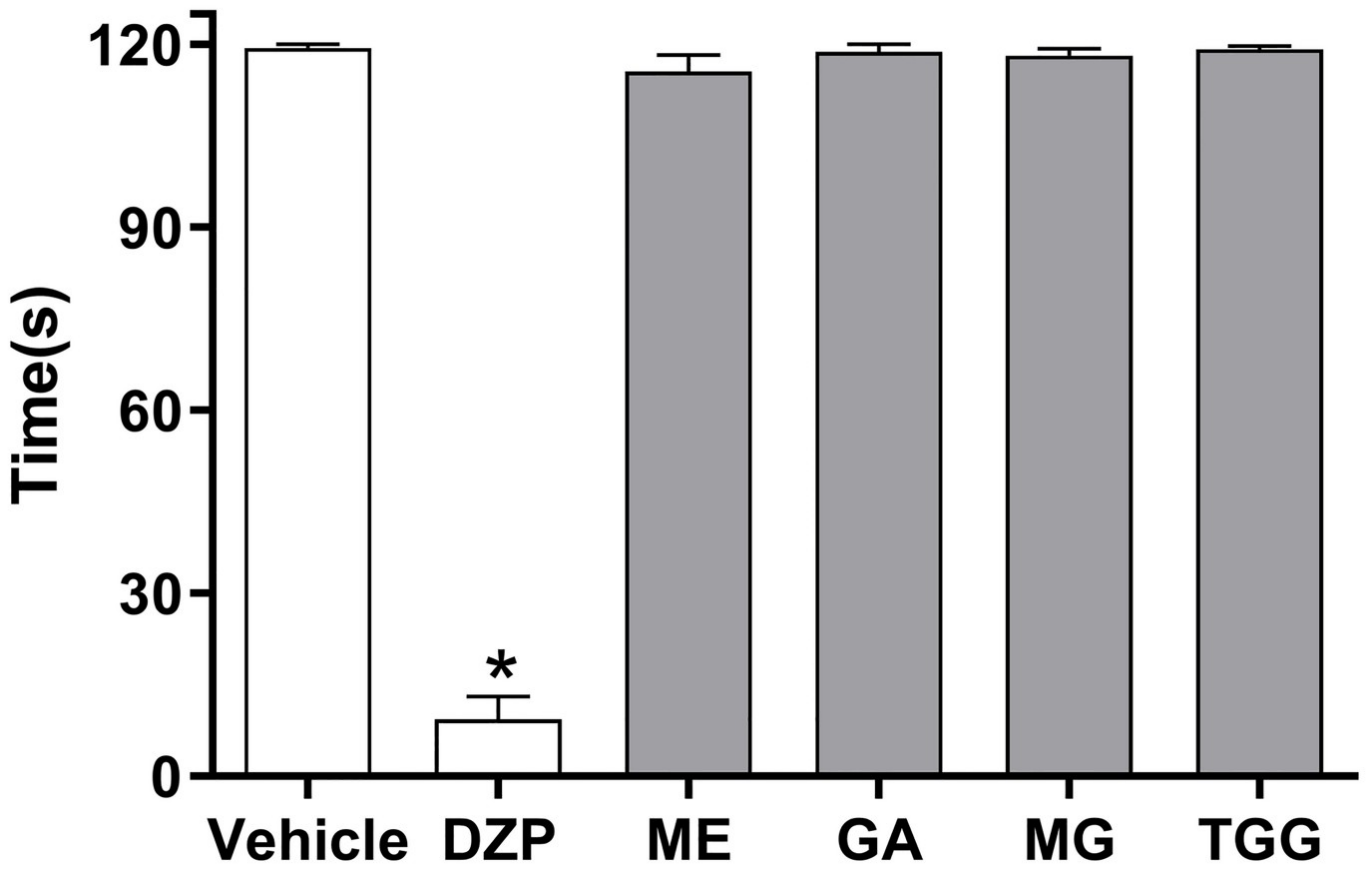
Effects of ME, gallic acid, methyl gallate and 1,2,6-tri-O-galloyl-β-d-glucopyranose from *D*. *vandellianum* on motor function in mice. Bar graph representing the run time on the rotarod, 40 min after intraplantar injection of ME (200 mg/kg), gallic acid (GA; 200 mg/kg), methyl gallate (MG; 200 mg/kg), 1,2,6-tri-O-galloyl-β-d-glucopyranose (TGG; 200 mg/kg), diazepam (DZP; 10 mg/kg, reference drug) or vehicle (saline; control group). Data are reported as means ± SEM; *n* = 6 mice per group. * Significantly different from the vehicle group (*p* < 0.001). One-way ANOVA followed by the Tukey’s test.

In the rotarod test, the intraperitoneal administration of ME, gallic acid, methyl gallate and 1,2,6-tri-O-galloyl-β-d-glucopyranose (200 mg/kg), did not reduce the run time of the mice, indicating that these treatments did not induce motor performance alterations (Fig 5). As expected, the central nervous system depressant diazepam (10 mg/kg) reduced the time of mice on the rotarod after 40 min of intraperitoneal treatment with this standard drug.

## Discussion

### Phytochemical profile

The 42 compounds were tentatively assigned in ethyl ether fraction from *D*. *vandellianum* by the interpretation of their fragmentation patterns obtained from mass spectra (HRESIMS, MS^2^ and MS^3^ experiments). Data provided by isolated compounds and literature information was also employed for the comprehensive evaluation of samples. The retention times and mass spectrum data along with peak assignments for compounds identified using negative ionization are described in Table 1.

Gallotannins and ellagitannins were detected and tentatively assigned. They were distinguished by their characteristic fragment ion spectra yielding sequential losses of galloyl (*m/z* 152), gallate (*m/z* 170), and phexahydroxydiphenic acid (HHDP) or ellagic acid residues (*m/z* 301). Gallotanins composed of one to four units of galloyl esters of glucose were identified. The galloylhexose derivatives (**1**, **5** and **6**) were assinalated based on the [M − H]^−^ ion at *m/z* 331 and MS/MS produced as typical product ions at *m/z* 271, 193, 169 and 125 [28, 29]. Five peaks (**8**, **9**, **12**, **13** and **17**) with the precursor ion at *m/z* 483 were assigned to digalloyl-hexoside relying on the MS and MS/MS spectra that showed product ions at *m/z* 331 [M−H−162]^−^ and 169 [M−H−162−152]^−^ corresponding to the neutral losses of hexose and galloyl moieties, respectively [28, 29]. Similarly, trigalloyl-hexoside (**15**, **19**, **23**, **27** and **29**) and tetragalloyl (**26**) were tentatively assignment [28, 29]. Furthermore, galloylquinic acid derivatives were identified from observation of the precursor’s ions at *m*/*z* 343, *m*/*z* 495 and *m*/*z* 647, which were attributed to galloylquinic acids (**4** and **7**), digalloylquinic acids (**11**, **14** and **18**) and trigalloylquinic acids (**22**) respectively. The MS^2^ and MS^3^ spectra indicated as products ions at *m*/*z* 191, 173, 169, 125 and 85, consistent with fragmentation behavior of galloylquinic acid derivatives. In addition, the compounds gallic acid (**3**), methytl gallate (**16**) and ethyl gallate (**24**) were identidied based on [M−H]^−^ions at *m/z* 169, 183 and 197, respectively. MS^2^ data of these ions were compared with literature data [28,29,31]. Ellagic acid (**38**) was identified based on the [M − H]^−^ ion at m/z 301 that was confirmed by MS^2^ spectrum with product ions at m/z 257, 229, 185 [34]. Moreover, others ellagitannins as chebulic acid and chebulagic acid were also identified [29].

Regarding to identification of flavonoids, they were classified in two groups: O-glycosides and C-glycosides, which were identified comparing their MS/MS spectra with those available in literature. It was observed two parent ions at m/z 301 and 285, both result as product an ion at m/z 151, identified as quercetin and kaempferol, respectively [28,35]. Besides parent ions at m/z 463 and 447 exhibited product ions at m/z 301 [M−H−162]^−^ and 285 [M−H−162]^−^, a characteristic loss of the hexose moiety, were identified as quercetin-O-hexoside (**35**) and kaempferol-O-hexoside (**39**), respectively [33]. Peaks **31** (m/z 615) and **36** (m/z 599) exhibited the same loss of 314Da characteristic of hexose and galloyl moieties, it originated fragment ions at m/z 301 and 285, which corresponds to quercetin and kaempferol. Thus, them were identified as galloylquercetin hexoside and astragalin-O-gallate, respectively [32,33]. Concerning to C-glycosides, fragments [M−H−60]^−^, [M−H−90]- and [M−H−120]^−^ work as characteristic diagnostic ions to glycine moiety. Peak **28** was identified as isoorientin, it presented [M−H]^−^ ion at m/z 447 and MS/MS spectra at m/z 429, 357, 327 and 285 [32]. Peak **33**, [M−H]^−^ ion at m/z 433, produced fragment ions at m/z 413, 341 and 311 corresponding to C-glycoside fragmentation pattern, which was suggested as isovitexin [32]. Peak **30** was identified as your isomer, vitexin [32].

Of the 42 compounds observed by HPLC-DAD-ESIMS^n^, three had their structures confirmed by NMR (gallic acid, methyl gallate, 1,2,6-tri-*O*-galloyl-β-d-glucopyranose).

Compound 1,2,6-tri-*O*-galloyl-β-d-glucopyranose was obtained as a yellowish brown amorphous powder. The ^1^H NMR spectrum reveals three singlets, each one integrating for two protons at *δ*_H_ 7.10, 7.15 and 7.18, assignable to the aromatic protons of the three galloyl moieties the molecule. In the carbohydrate region, the spectrum clearly shows three downfield hydrogens resonances: a doublet at *δ*_H_ 5.85 integrating one proton with large coupling constant, indicating a *β*-configuration of the anomeric proton; a triplet at *δ*_H_ 5.31, and two signals at *δ*_H_ 4.61 (d, *J* = 12 Hz) and 4.47 (dd, *J* = 12, 4.4 Hz) which were assigned to H-2 and H-6 glucose hydrogen [38]. The signals of these hydrogens are downfield compared to those of β-d-glucopyranose, indicating the location of galloyl units at these positions [39]. The structure was confirmed by the analysis of the ^13^C-NMR spectrum. Three carbonyl ester signals at *δ*_C_ 166.9, 168.2 and 168.3, confirm the presence of the three galloyl moieties. Signals at *δ*_C_ 94.5 (C-1), 68.3 (C-2) and 62.9 (C-6) indicate that the hydroxyl groups at these positions are galloylated [35]. Its molecular formula was defined as C_27_H_24_O_18_ by HRESIMS at *m*/*z* 635.0890 [M − H]^−^ (calculated for C_27_H_23_O_18_, 635.0879, Δ = −1.7 ppm). The structure of this gallotannin was confirmed by comparison of the above described spectra data with the literature and was identified as 1,2,6-tri-*O*-galloyl-β-d-glucopyranose [40].

The isolated compounds gallic acid and methyl gallate also had their structures confirmed by comparing the ^1^H and ^13^C NMR and mass spectrometry data with the literature data [40–42]. In such a way, the known compounds gallic acid, methyl gallate, 1,2,6-tri-*O*-galloyl-β-d-glucopyranose were identified.

### Antinociceptive activity

The present study demonstrates that systemic administration of methanol extract of *D*. *vandellianum* leaves produces a consistent antinociceptive effect. Fractionation of *D*. *vandellianum* leaves showed that this effect could be attributed, at least in part, to their bioactive constituents gallic acid, methyl gallate and 1,2,6-tri-O-galloyl-β- d-glucopyranose.

The formalin test is a useful screening tool for assessment of the analgesic properties of plant extracts and compounds. In the present study, data from this assay indicated that ME induces a dose-dependent antinociceptive effect. Afterwards, this methanol extract was fractionated and the obtained fractions (ether, ethyl acetate and chloroform) were also evaluated in formalin test. All tested fractions presented antinociceptive effects *in vivo*, but the ethyl ether fraction was the most active. In line with this result, ethyl ether fraction was selected for the phytochemical study, evidencing three major compounds: gallic acid, methyl gallate and 1,2,6-tri-O-galloyl-β-d-glucopyranose. In order to evaluate the eventual contribution of these compounds to antinociceptive action of ME, their biological properties were next evaluated in the formalin test.

The results obtained from the present work demonstrate that GA induces antinociceptive effects in the late phase of the formalin test. From a pharmacological point of view, it has been proposed that drugs that block the nociception transmission, such as opioid analgesics, suppress both phases of formalin-induced pain response, whereas nonsteroidal anti-inflammatory drugs, such as indomethacin, seem to suppress only the late phase [43]. Based on these concepts, it is possible to propose that GA-induced antinociception is associated with anti-inflammatory properties. In line with this idea, the antinociceptive effect of GA has been demonstrated in the carrageenan-induced inflammatory pain model [44]. In addition, this compound has also been reported to have relevant anti-inflammatory and immunomodulatory activities [45–47].

Like GA, methyl gallate presented an antinociceptive effect on the inflammatory phase of formalin test, displaying analgesic activity with a similar profile of NSAIDS. Supporting this hypothesis, it was previously demonstrated that MG has anti-inflammatory and cyclooxygenase-2 inhibitory activities [48,49]. Even though the antinociceptive properties of gallic acid ethyl ester have been previously described [50], the present data demonstrate the antinociceptive effect of MG.

Few studies have reported the pharmacological properties of TGG, and most of them describe its antibacterial and antiviral activities [40,51–53]. A previous study has demonstrated antinociceptive activity of methanolic extract of *Miconia minutiflora* (Bonpl.) DC., which contains gallotannin class in its composition [54]. The present study displays the direct pharmacological evidence for the antinociceptive activity of gallotannin. TGG exhibited antinociceptive effect on the inflammatory phase of formalin test, and was more efficacious than indomethacin, the analgesic anti-inflammatory drug used as a gold standard in this test. Corroborating this relevant effect of TGG on the inflammatory phase of formalin, Erdèlyi and coworkers demonstrated by *in vitro* experiments, that this class of compound is able to reduce the inflammatory cytokines expression [55]. Importantly, gallic acid, methyl gallate and 1,2,6-tri-O-galloyl-β-d-glucopyranose at antinociceptive doses did not affect the motor performance in mice in the rotarod test. These results corroborate the antinociceptive properties pointed by formalin test.

Despite consistent antinociceptive effects induced by systemic treatment with GA, MG and TGG, the contribution of others bioactive molecules to ME-induced antinociception cannot be ruled out. The ether ethyl fraction of ME presented 39 minor compounds, and it is well accepted that minor constituents in plant extracts may contribute to pharmacological properties of these extracts through synergistic actions or independent pharmacological effects [56]. In addition, among the minor compounds identified in ME, some of them, such as kaempferol and quercetin, have well-stablished antinociceptive activity [57,58]. Antinociceptive properties of quercetin have been demonstrated in both experimental and clinical conditions. Quercetin produces dose-related antinociception in several models of chemical pain, such as acetic acid test, formalin test, nociception induced by glutamate and capsaicin [57], and oxaliplatin-induced neuropathic pain [9], showing also analgesic properties in clinical conditions [59]. Similarly, kaempferol treatment is found to attenuate neuropathic pain [60] and chemical pain [61] in pre-clinical studies. Thus, it is possible that these compounds contribute to the antinociceptive effect of the ether ethyl fraction of ME, however, this hypothesis has not been investigated here.

## Conclusions

The present study, using a classic model of analgesic drug screening combined with reliable methods of structural analysis, demonstrated that the methanolic extract of *D*. *vandellianum* leaves shows antinociceptive properties on experimental inflammatory pain. The isolation indicated that gallic acid, methyl gallate and 1,2,6-tri-O-galloyl-β-d-glucopyranose, major constituents of the ether fraction from ME, are the antinociceptive components of *D*. *vandellianum*. The antinociceptive properties demonstrated in formalin test were corroborated by the results of rotarod test, which did not demonstrate motor deficits and nonspecific depression in the nervous system. To the best of our knowledge this is the first report of the antinociceptive activity of 1,2,6-tri-O-galloyl-β-d-glucopyranose. The occurrence of these compounds in *D*. *vandellianum* is also described for the first time.

## References

[1] Sarmento-Neto JF, do Nascimento LG, Felipe CF, de Sousa DP. Analgesic Potential of Essential Oils. Molecules. 2015; 21: 20. 10.3390/molecules21010020 PMC6273222 26703556

[2] Li JX. Combining opioids and non-opioids for pain management: Current status. Neuropharmacology. 2019; 10.1016/j.neuropharm.2019.04.025. 31029588

[3] Turk DC, Wilson HD, Cahana A. Treatment of chronic non-cancer pain. Lancet. 2011; 377: 2226–2235. 10.1016/S0140-6736(11)60402-9 21704872

[4] Calixto JB, Beirith A, Ferreira J, Santos AR, Filho VC, Yunes RA. Naturally occurring antinociceptive substances from plants. Phytother. Res. 2000; 6: 401–418. 10.1002/1099-1573(200009)14:6<401::aid-ptr762>3.0.co;2-h 10960893

[5] Sen S, Chakraborty R, De B, Ganesh T, Raghavendra HG, Debnath S. Analgesic and anti-inflammatory herbs: A potential source of modern medicine. Int. J. Pharm. Sci. Res. 2010; 1: 32–44.

[6] De Almeida Barros TA, De Freitas LAR, Filho JMB, Nunes XP, Giulietti AM, De Souza GE, et al Antinociceptive and anti-inflammatory properties of 7-hydroxycoumarin in experimental animal models: Potential therapeutic for the control of inflammatory chronic pain. J. Pharm. Pharmacol. 2010; 62: 205–213. 10.1211/jpp.62.02.0008 20487200

[7] De Lima FO, Nonato FR, Couto RD, Barbosa Filho JM, Nunes XP, et al Mechanisms involved in the antinociceptive effects of 7-hydroxycoumarin. J. Nat. Prod. 2011; 74: 596–602. 10.1021/np100621c 21417376

[8] Espírito-Santo RF, Meira CS, Costa RdS, Souza Filho OP, Evangelista AF, Trossini GHG, et al The anti-inflammatory and immunomodulatory potential of braylin: Pharmacological properties and mechanisms by *in silico*, *in vitro* and *in vivo* approaches. PLoS ONE. 2017; 12(6): e0179174 10.1371/journal.pone.0179174 28594906 PMC5464642

[9] Azevedo MI, Pereira AF, Nogueira RB, Rolim FE, Brito GAC, Wong DVT, et al The antioxidant effects of the flavonoids rutin and quercetin inhibit oxaliplatin-induced chronic painful peripheral neuropathy. Mol. Pain. 2013; 9: 53–53. 10.1186/1744-8069-9-53 24152430 PMC3835704

[10] Higgs J, Wasowski C, Loscalzo LM, Marder M. In vitro binding affinities of a series of flavonoids for μ-opioid receptors. Antinociceptive effect of the synthetic flavonoid 3,3-dibromoflavanone in mice. Neuropharmacology. 2013; 72: 9–19. 10.1016/j.neuropharm.2013.04.020 23624290

[11] De Queiroz AC, Alves HDS, Cavalcante-Silva LHA, Dias TDLMF, Santos MDS, Melo GMDA, et al Antinociceptive and anti-inflammatory effects of flavonoids PMT1 and PMT2 isolated from Piper montealegreanum Yuncker (Piperaceae) in mice. Nat. Prod. Res. 2014; 28: 403–406. 10.1080/14786419.2013.867444 24479832

[12] Waterman PG. Alkaloids of the rutaceae: Their distribution and systematic significance. Biochem. Syst. Ecol. 1975; 3: 149–180.

[13] Vieira PC, Lazaro AR, Fernandes JB, Da Silva MFDGF. Limonoids, alkaloids, and chromones from Dictyoloma vandellianum, and their chemosystematic significance. Quim. Nova. 1990; 13: 287–288.

[14] Campos AM, Khac DD, Fetizon M. Chromones from Dictyoloma incanescens. Phytochemistry. 1987; 26: 2819–2823.

[15] Vieira PC, Lazaro AR, Fernandes JB, Da Silva MFDGF. The chemosystematics of Dictyoloma. Biochem. Syst. Ecol. 1988; 16: 541–544.

[16] Sartor CFP, Da Silva MFDGF, Fernandes JB, Vieira PC, Fo ER, Garcia Cortez DA. Alkaloids from Dictyoloma vandellianum: Their chemosystematic significance. Phytochemistry. 2003; 63: 185–192. 10.1016/s0031-9422(03)00006-2 12711140

[17] Alves IM, Abreu LS, Costa CO, Le Hyaric M, Guedes ML, Soares MB, et al Pyranochromones from Dictyoloma vandellianum A. Juss and Their Cytotoxic Evaluation. Chem. Biodiversity. 2017; 14: e1600276. 10.1002/cbdv.201600276 27797447

[18] Lavaud C, Massiot G, Vasquez C, Moretti C, Sauvain M, Balderrama L. 4-Quinolinone alkaloids from Dictyoloma peruviana. Phytochemistry. 1995; 40: 317–320. 10.1016/0031-9422(95)00265-9 7546553

[19] Michael JP. Quinoline, quinazoline and acridone alkaloids. Nat. Prod. Rep. 1997; 14: 11–20. 9121729 10.1039/np9971400011

[20] Roy A, Saraf S. Limonoids: Overview of significant bioactive triterpenes distributed in plants kingdom. Biol. Pharm. Bull. 2006; 29: 191–201. 10.1248/bpb.29.191 16462017

[21] Heeb S, Fletcher MP, Chhabra SR, Diggle SP, Williams P, Cámara M. Quinolones: From antibiotics to autoinducers. Fems. Microbiol. Rev. 2011; 35: 247–274. 10.1111/j.1574-6976.2010.00247.x 20738404 PMC3053476

[22] Sharma S, Kumar S, Chand K, Kathuria A, Gupta A. An update on natural occurrence and biological activity of chromones. Curr. Med. Chem. 2011; 18: 3825–3852. 10.2174/092986711803414359 21824102

[23] Opretzka LCF, Espírito-Santo RF, Nascimento OA, Abreu LS, Alves IM, Döring E, et al Natural chromones as potential anti-inflammatory agents: Pharmacological properties and related mechanisms. Int. Immunopharm. 2019; 72: 31–39. 10.1016/j.intimp.2019.03.044 30959369

[24] Dubuisson D, Dennis SG. The formalin test: a quantitative study of the analgesic effects of morphine, meperidine, and brain stem stimulation in rats and cats. Pain. 1977; 4: 161–174. 10.1016/0304-3959(77)90130-0 564014

[25] Tjolsen A, Berge OG, Hunskaar S, Rosland JH, Hole K. The formalin test: an evaluation of the method. Pain. 1992; 51: 5–17. 10.1016/0304-3959(92)90003-t 1454405

[26] Hunskaar S, Hole K. The formalin test in mice: Dissociation between inflammatory and non-inflammatory pain. Pain. 1987; 30: 103–114. 10.1016/0304-3959(87)90088-1 3614974

[27] Shibata M, Ohkubo T, Takahashi H, Inoki R. Modified formalin test: Characteristic biphasic pain response. Pain. 1989; 38: 347–352. 10.1016/0304-3959(89)90222-4 2478947

[28] Metzker De Oliveira C, Nonato FR, Oliveira De Lima F, Couto RD, David JP, David JM, et al Antinociceptive properties of bergenin. J. Nat. Prod. 2011; 74: 2062–2068. 10.1021/np200232s 21939182

[29] Gama KB, Quintans JSS, Antoniolli AR, Quintans L.J Jr, Santana WA, Branco A, et al Evidence for the involvement of descending pain-inhibitory mechanisms in the antinociceptive effect of hecogenin acetate. J. Nat. Prod. 2013; 76: 559–563. 10.1021/np3007342 23437926

[30] Abu-Reidah IM, Ali-Shtayeh MS, Jamous RM, Arráez-Román D, Segura-carretero A. HPLC–DAD–ESI-MS/MS screening of bioactive components from Rhus coriaria L.(Sumac) fruits. Food Chem. 2015; 166: 179–191. 10.1016/j.foodchem.2014.06.011 25053044

[31] Yang B, Kortesniemi M, Liu P, Karonen M, Salminen JP. Analysis of hydrolyzable tannins and other phenolic compounds in emblic leafflower (Phyllanthus emblica L.) fruits by high performance liquid chromatography–electrospray ionization mass spectrometry. J. Agr. Food Chem. 2012; 60: 8672–8683. 22889097 10.1021/jf302925v

[32] Clifford MN, Stoupi S, Kuhnert N. Profiling and characterization by LC-MS n of the galloylquinic acids of green tea. tara tannin. and tannic acid. J. Agr. Food Chem. 2007; 5: 2797–2807. 10.1021/jf063533l 17381119

[33] Chen H, Li M, Zhang C, Du W, Shao H, Feng Y, et al Isolation and Identification of the Anti-Oxidant Constituents from Loropetalum chinense (R. Brown) Oliv. Based on UHPLC⁻Q-TOF-MS/MS. Molecules. 2018; 23: 1720. 10.3390/molecules23071720 PMC6099825 30011908

[34] Lin YL, Kuo YH, Shiao MS, Chen CC, Ou JC. Flavonoid glycosides from Terminalia catappa L. J. Chinese Chem. Soci. 2000; 47: 253–256.

[35] Fraige K, Dametto AC, Zeraik ML, de Freitas L, Saraiva AC, Medeiros AI, et al Dereplication by HPLC-DAD-ESI-MS/MS and Screening for Biological Activities of Byrsonima Species (Malpighiaceae). Phytochem. Anal. 2018; 29:196–204. 10.1002/pca.2734 28990237

[36] Lee JH, Johnson JV, Talcott ST. Identification of ellagic acid conjugates and other polyphenolics in muscadine grapes by HPLC-ESI-MS. J. Agr. Food Chem. 2005; 53: 6003–6010. 16028988 10.1021/jf050468r

[37] Chen G, Li X, Saleri F, Guo M. Analysis of flavonoids in rhamnus davurica and its antiproliferative activities. Molecules. 2016; 21: 1275. 10.3390/molecules21101275 PMC6273673 27669205

[38] Nawwar MAM, Hussein SAM. NMR spectra analysis of polyphenols from Punica granatum. Phytochem. 1994; 36: 793–798.

[39] De Bruyn A, Anteunis M, Van Beeumen J. Chemical shifts of aldohexopyranoses revisited and application to gulosylglucose. Bull. Soc. Chim. Belg. 1977; 86: 259–265.

[40] Bag A, Bhattacharyya SK, Chattopadhyay RR. Isolation and identification of a gallotannin 1,2,6-tri-O-galloyl-β-d-glucopyranose from hydroalcoholic extract of Terminalia chebula fruits effective against multidrug-resistant uropathogens. J. Appl. Microbiol. 2013; 115: 390–397. 10.1111/jam.12256 23683054

[41] Chanwitheesuk A, Teerawutgulrag A, Kilburn JD, Rakariyatham N. Antimicrobial gallic acid from Caesalpinia mimosoides Lamk. Food Chem. 2006; 100: 1044–1048.

[42] Wang CR, Zhou R, Ng TB, Wong JH, Qiao WT, Liu F. First report on isolation of methyl gallate with antioxidant, anti-HIV-1 and HIV-1 enzyme inhibitory activities from a mushroom (Pholiota adiposa). Environ. Toxicol. Pharmacol. 2014; 37: 626–637. 10.1016/j.etap.2014.01.023 24572641

[43] Le BD, Gozariu M, Cadden SW. Animal models of nociception. Pharmacol. Rev. 2001; 53: 597–652. 11734620

[44] Trevisan G, Rossato MF, Tonello R, Hoffmeister C, Klafke JZ, Rosa F, et al Gallic acid functions as a TRPA1 antagonist with relevant antinociceptive and antiedematogenic effects in mice. Naunyn-Schmiedeberg’s Arch Pharmacol. 2014; 387: 679–689. 24722818 10.1007/s00210-014-0978-0

[45] Kroes BH, Van Den Berg AJJ, Quarles Van Ufford HC, Van Dijk H, Labadie RP. Anti-inflammatory activity of gallic acid. Planta. Med. 1992; 58: 499–504. 10.1055/s-2006-961535 1336604

[46] Deng H, Fang Y. Anti-inflammatory gallic acid and wedelolactone are G protein-coupled receptor-35 agonists. Pharmacology. 2012; 89: 211–219. 10.1159/000337184 22488351

[47] Hsiang CY, Hseu YC, Chang YC, Kumar KJS, Ho TY, Yang HL. Toona sinensis and its major bioactive compound gallic acid inhibit LPS-induced inflammation in nuclear factor-κB transgenic mice as evaluated by in vivo bioluminescence imaging. Food Chem. 2013; 136: 426–434. 10.1016/j.foodchem.2012.08.009 23122080

[48] Kim SJ, Jin M, Lee E, Moon TC, Quan Z, Yang JH, et al Effects of methyl gallate on arachidonic acid metabolizing enzymes: Cyclooxygenase-2 and 5-lipoxygenase in mouse bone marrow-derived mast cells. Arch Pharmacal. Res. 2006; 29: 874–878. 10.1007/BF02973908 17121182

[49] Correa LB, Pádua TA, Seito LN, Costa TE, Silva MA, Candéa AL, et al Anti-inflammatory effect of methyl gallate on experimental arthritis: Inhibition of neutrophil recruitment, Production of inflammatory mediators, and activation of macrophages. J. Nat. Prod. 2016; 79: 1554–1566. 10.1021/acs.jnatprod.5b01115 27227459

[50] Santos AR, De Campos RO, Miguel OG, Cechinel-FilhO V, Yunes RA, Calixto JB. The involvement of K^+^ channels and Gi/o protein in the antinociceptive action of the gallic acid ethyl ester. Eur. J. Pharmacol. 1999; 379: 7–17. 10.1016/s0014-2999(99)00490-2 10499367

[51] Duan D, Li Z, Luo H, Zhang W, Chen L, Xu X. Antiviral compounds from traditional Chinese medicines Galla Chinese as inhibitors of HCV NS3 protease. Bioorg. Med. Chem. Lett. 2004; 14: 6041–6044. 10.1016/j.bmcl.2004.09.067 15546725

[52] Bag A, Chattopadhyay RR. Efflux pump inhibitory activity of a gallotannin from Terminalia chebula fruit against multidrug-resistant uropathogenic Escherichia coli. Nat. Pro. Res. 2014; 28: 1280–1283. 10.1080/14786419.2014.895729 24620744

[53] Bag A, Chattopadhyay RR. Synergistic antibiofilm efficacy of a gallotannin 1,2,6-tri-O-galloyl-β-d-glucopyranose from *Terminalia chebula* fruit in combination with gentamicin and trimethoprim against multidrug resistant uropathogenic *Escherichia coli* biofilms. PLoS ONE 2017; 12: e0178712 10.1371/journal.pone.0178712 28562631 PMC5451073

[54] Gatis-Carrazzoni ASSG, Mota FVB, Leite TCC. Anti-inflammatory and antinociceptive activities of the leaf methanol extract of Miconia minutiflora (Bonpl.) DC. and characterization of compounds by UPLC-DAD-QTOF-MS/MS. Naunyn-Schmiedeberg’s Arch Pharmacol. 2019; 392: 55–68. 30215112 10.1007/s00210-018-1561-x

[55] Erdelyi K, Kiss A, Bakondi E, Bai P, Szabo C, Gergely P, et al Gallotannin inhibits the expression of chemokines and inflammatory cytokines in A549 cells. Mol. Pharmacol. 2005; 68: 895–904. 10.1124/mol.105.012518 15976037

[56] Wagner H, Ulrich-Merzenich G. Synergy research: Approaching a new generation of phytopharmaceuticals. Phytomed. 2009; 16: 97–110. 10.1016/j.phymed.2008.12.018 19211237

[57] Filho AW, Filho VC, Olinger L, De Souza MM. Quercetin: Further investigation of its antinociceptive properties and mechanisms of action. Arch. Pharm. Res. 2008; 31: 713–721. 10.1007/s12272-001-1217-2 18563352

[58] Maleki-Dizaji N, Fathiazad F, Garjani A. Antinociceptive properties of extracts and two flavonoids isolated from leaves of Danae racemosa. Arch. Pharmacal. Res. 2007; 30: 1536–1542. 10.1007/BF02977322 18254240

[59] Javadi F, Ahmadzadeh A, Eghtesadi S, Aryaeian N, Zabihiyeganeh M, Rahimi Foroushani A, Jazayeri S. The Effect of Quercetin on Inflammatory Factors and Clinical Symptoms in Women with Rheumatoid Arthritis: A Double-Blind, Randomized Controlled Trial. J Am Coll Nutr. 2017; 36: 9–15. 10.1080/07315724.2016.1140093 27710596

[60] Abu-Salem OM. Kaempferol attenuates the development of diabetic neuropathic pain in mice: possible anti-inflammatory and anti-oxidantmechanisms. Maced J. Med. 2014; 7(3): 424–430.

[61] De Melo GO, Malvar DC, Vanderlinde FA, Rocha FF, Pires PA, Costa EA, et al Antinociceptive and anti-inflammatory kaempferol glycosides from Sedum dendroideum. J. Ethnopharmacol. 2009; 124: 228–232. 10.1016/j.jep.2009.04.024 19397977

